# Novel Biomarker MicroRNAs for Subtyping of Acute Coronary Syndrome: A Bioinformatics Approach

**DOI:** 10.1155/2016/4618323

**Published:** 2016-12-01

**Authors:** Yujie Zhu, Yuxin Lin, Wenying Yan, Zhandong Sun, Zhi Jiang, Bairong Shen, Xiaoqian Jiang, Jingjing Shi

**Affiliations:** ^1^Center for Systems Biology, Soochow University, Suzhou 215006, China; ^2^Biomedical Informatics Division, UC San Diego, La Jolla, CA, USA; ^3^Nanjing Drum Tower Hospital, The Affiliated Hospital of Nanjing University Medical School, Nanjing, Jiangsu 210008, China; ^4^School of Medicine, Soochow University, Suzhou 215123, China; ^5^Department of Cardiovascular Internal Medicine, Wuxi Third People's Hospital, Wuxi 214041, China

## Abstract

Acute coronary syndrome (ACS) is a life-threatening disease that affects more than half a million people in United States. We currently lack molecular biomarkers to distinguish the unstable angina (UA) and acute myocardial infarction (AMI), which are the two subtypes of ACS. MicroRNAs play significant roles in biological processes and serve as good candidates for biomarkers. In this work, we collected microRNA datasets from the Gene Expression Omnibus database and identified specific microRNAs in different subtypes and universal microRNAs in all subtypes based on our novel network-based bioinformatics approach. These microRNAs were studied for ACS association by pathway enrichment analysis of their target genes. AMI and UA were associated with 27 and 26 microRNAs, respectively, nine of them were detected for both AMI and UA, and five from each subtype had been reported previously. The remaining 22 and 21 microRNAs are novel microRNA biomarkers for AMI and UA, respectively. The findings are then supported by pathway enrichment analysis of the targets of these microRNAs. These novel microRNAs deserve further validation and will be helpful for personalized ACS diagnosis.

## 1. Introduction

Acute coronary syndrome (ACS) is caused by decreased blood flow in the coronary arteries arising from thrombus formation and possible coronary vasospasm, which may further lead to heart muscle dysfunction or even death [1]. In 2010, it was estimated that the number of hospital discharges with ACS was 625,000 in the United States, and secondary discharge diagnoses showed the number of inpatient hospital discharges was 1,141,000 for ACS [2]. The death toll range from ACS is the same as sepsis [3]. ACS is not only one of the severest diseases but also an economic burden to society, costing Americans more than 150 billion dollars annually [4]. The two subtypes of ACS are unstable angina (UA) (38%) and acute myocardial infarction (AMI), including ST-elevation myocardial infarction (30%) and non-ST-elevation myocardial infarction (25%) [5].

Over the past few decades, patients were usually checked with an initial evaluation and assessed with a risk score or prediction algorithms considering clinical history, physical examination, and other indices [6–9]. Additional tests have been added to these assessments including electrocardiogram [10], coronary computed tomographic angiography [11], muscle and brain fraction of creatine kinase [12], or blood tests such as troponin I or T [13]. However, the current methods are insufficient for a highly sensitive and specific diagnosis, especially in distinguishing AMI from UA. In addition, there is a phenomenon called “silent” myocardial infarction, which is estimated to occur in around 64% of cases, in which patients do not have chest pain or other symptoms [14]. It is therefore urgent to discover more effective biomarkers to precisely diagnosis the subtypes of ACS.

MicroRNAs (miRNAs) are a class of small noncoding RNAs with the posttranscriptional role of regulating about 60% of human protein-coding genes [15]. Currently there are more than 2500 mature human miRNAs listed in miRBase (release 21) [16]. They play functions in a wide variety of biological processes such as cell proliferation [17, 18], development [19], and apoptosis [20], which contribute to various physiological and pathological conditions, including cardiovascular diseases such as the acute coronary syndrome [21, 22].

**Figure 1:**
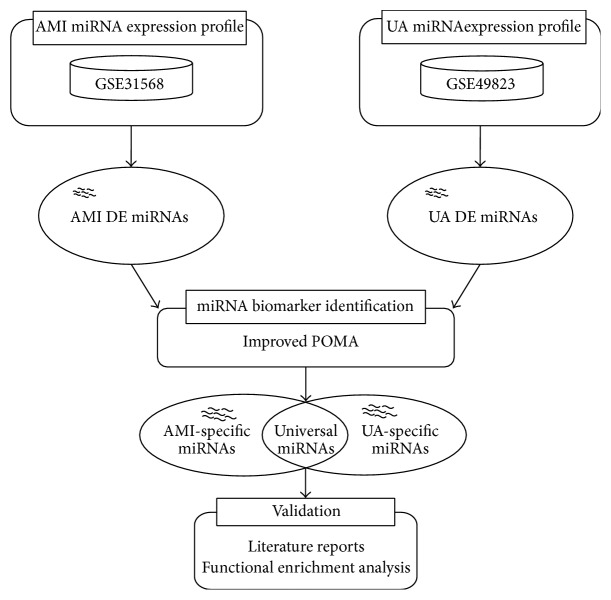
Schematic diagram for the identification of candidate miRNA biomarkers in acute myocardial infarction (AMI) and unstable angina (UA). Here, “DE” is the abbreviation of “differentially expressed.”

Until now, very few studies have looked at the two ACS subtypes, AMI and UA, in terms of similarities and differences, and in particular miRNA expression levels have not been well studied. To better understand the disease pathogenesis of these two subtypes, we applied an in-house regulatory model termed improved Pipeline of Outlier MicroRNA Analysis (POMA) [23, 24] to identify miRNAs specific to each subtype or shared by both subtypes (see Figure 1). The model focused on miRNAs' independent regulatory power and the biological functions of their targets. Two measures, novel out degree (NOD) and transcription factor percentage (TFP) of genes, were defined, where NOD was equivalent to the number of genes that were uniquely targeted by a single miRNA and TFP represented the percentage of all transcription factor (TF) genes that were targeted. According to the statistical evidences described in our previous work, miRNAs with larger NOD and TFP values were more likely to be candidate biomarkers and represented biomarker miRNAs that had strong abilities to regulate genes independently and, meanwhile, regulate more TF genes. The application of biomarker discovery for prostate cancer [23, 25], sepsis [26], clear cell renal cell carcinoma [27], and pediatric acute myeloid leukemia [24] demonstrated its great predictive power.

## 2. Materials and Methods 

### 2.1. Dataset Collection

The miRNA expression datasets (GSE31568 and GSE49823) were downloaded from Gene Expression Omnibus (GEO) [28]. GSE31568 contained 454 samples and we extracted 70 controls and 21 AMI samples [29], and GSE49823 contained 13 controls and 13 UA samples. The details of the two datasets are listed in Table 1. We then identified differentially expressed (DE) miRNAs based on linear models in Limma R package [30, 31]; the empirical Bayes (eBayes) method was performed to calculate the *p* value and other parameters. The Benjamini-Hochberg method was applied to adjust and correct *p* values. The adjusted *p* value <0.05 was chosen as the cut-off criteria.

We also collected the reported miRNAs for AMI and UA from PubMed by the search criteria “(Acute Myocardial Infarction OR AMI) AND (miRNA OR microRNA) AND (biomarker^*∗*^ OR marker^*∗*^)” and “(Unstable Angina OR UA) AND (miRNA OR microRNA) AND (biomarker^*∗*^ OR marker^*∗*^)”. We only took published reports from the past five years and all of the samples were extracted with human data in consideration. The information of biomarkers including miRNA ID, biomarker type, expression pattern, study design, publication date, and PMID are summarized in Tables S1 and S2, in Supplementary Material available online at http://dx.doi.org/10.1155/2016/4618323.

### 2.2. Prediction of Putative miRNA Biomarkers for AMI and UA

Based on two significantly DE miRNA sets, we employed improved POMA to predict miRNA biomarkers for AMI and UA [24]. In the pipeline, two important measures NOD and TFP were defined. NOD is the number of genes uniquely targeted by a certain miRNA and TFP is the percentage of TF genes of all targets of the miRNA. The main idea of the improved POMA model is that miRNAs with larger NOD values and targeting more TF genes are more likely to be biomarkers. The POMA and improved POMA methodologies were elaborated in our previous studies [23, 24].

Using this pipeline, the AMI- and UA-specific miRNA-mRNA networks were constructed by mapping relevant DE miRNAs onto human miRNA-mRNA network (reference network). Then, NOD and TFP were measured for each miRNA in the condition-specific network of AMI and UA, respectively. Finally, miRNAs with significantly large NOD and TFP values (Wilcoxon signed-rank test, *p* value <0.05) were selected as candidate biomarkers.

We calculated the percentage of reported AMI/UA biomarker miRNAs in the whole predicted set and defined it as the prediction precision for evaluating the accuracy of our model.

### 2.3. Functional Enrichment Analysis of the Target Genes of Candidate miRNA Biomarkers

We performed functional enrichment analysis of the genes uniquely regulated by candidate biomarker miRNAs from the two condition-specific miRNA-mRNA networks by MetaCore™ software. The significantly enriched pathways and diseases ontologies were ranked by *p* value (<0.05), which was calculated by hypergeometric test. FDR adjustment was used for multiple test correction.

## 3. Results

### 3.1. Identification of Candidate miRNA Biomarkers for AMI and UA

**Figure 2:**
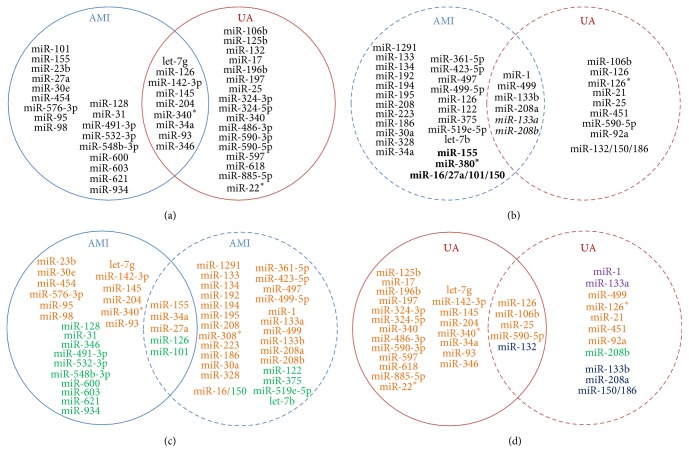
The Venn diagram of miRNA biomarkers for acute myocardial infarction (AMI) and unstable angina (UA). Circles in blue and red represent miRNAs for AMI and UA, respectively. Solid and dashed lines represent identified and literature reported miRNAs, respectively. (a) Candidate miRNA biomarkers identified by our model. (b) Biomarker miRNAs collected from published literature. IDs in bold mean they were prognostic, those in italic meant they were functional for both diagnosis and prognosis, and the remaining ones were reported to be diagnostic markers. (c) Comparison of AMI miRNA biomarkers identified by our model and published literature. IDs in orange and green represent up- and downregulated miRNAs, respectively. (d) Comparison of UA miRNA biomarkers identified by our model and published literature. IDs in orange and green represent up- and downregulated miRNAs, respectively. miRNAs that had both up- and downexpression patterns are colored in purple, and those with unclear expression patterns are colored in dark blue.

Based on AMI and UA miRNA expression datasets, we identified 292 and 182 deregulated miRNAs in AMI and UA, respectively. Employing our in-house model improved POMA [24], and a total of 27 miRNAs for AMI and 26 miRNAs for UA were screened (see Figure 2(a), Wilcoxon signed-rank test, *p* value < 0.05). These miRNAs were predicted to be candidate biomarkers for the two subtypes of ACS by our model. The substructural characteristics of these biomarker miRNAs in the miRNA regulatory network, including the number of whole targets (termed *N*), NOD, and TFP values, are listed in Table 2.

As listed in Table 2, nine miRNA biomarkers were shared by both AMI and UA subtypes, indicating that these miRNAs (miR-126, miR-142-3p, miR-145, miR-204, miR-340^*∗*^, miR-346, miR-34a, miR-93, and let-7g) could be universal biomarkers for both AMI and UA. The remaining 18 and 17 miRNAs could be putative biomarkers specific for AMI and UA, respectively.

### 3.2. Literature-Based Validation of Identified miRNA Biomarkers

We collected AMI- and UA-specific miRNA biomarkers by analysis of citations in PubMed, as shown in Figure 2(b). Altogether, 30 miRNAs have been reported to be biomarkers for AMI and 25 of them are diagnostic. Two miRNAs (miR-155 and miR-380^*∗*^) [32] and a cluster of miR-16/27a/101/150 [33] were reported to be prognostic indicators. Two miRNAs (miR-208b and miR-133a) were reported to be valuable for both diagnosis and prognosis in AMI (see Table S1).

For UA, 15 miRNAs have been reported to be biomarkers, 13 of them were diagnostic, including a cluster of three miRNAs (miR-132/150/186) [34], and two were reported to be effective for both diagnosis and prognosis (miR-133a and miR-208b) [35] (see Table S2). We then compared literature reported miRNAs with ones we identified and found five that were the same in the AMI set (prediction precision: 18.5%): miR-155, miR-34a, miR-27a, miR-101, and miR-126 (see Figure 2(c)). Among them, miR-155 expression was increased approximately 4-fold in patients with a high-risk of cardiac death after discharge and could be a biomarker for cardiac death in post-AMI patients [32]; miR-34a was investigated for its role as a p53 responsive miRNA and confirmed as predicator for the risk of heart failure after AMI [36]. Elevated miR-27a expression was included in the panel of prognostic miRNAs for outcome after AMI; downregulation of miR-101 was also included in this panel. However, miR-101 was also reported to be upregulated in another study [33].

There were also five biomarker miRNAs (miR-106b, miR-25, miR-590-5p, miR-132, and miR-126) for UA found from our analysis and the reported list (see Figure 2(d), prediction precision: 19.2%). Among them, miR-106b, miR-25, and miR-590-5p were upregulated when compared with the control group [37]. The significantly elevated expression levels of the miR-106b/25 cluster and miR-21/590-5p family could be used as an indicator of coronary artery disease. A panel that consisted of miR-132, miR-150, and miR-186 showed the highest discriminatory power (AUC = 0.91) [34]. miR-126 was a unique biomarker that was found both in our analysis and in previous studies for both AMI and UA. However, miR-126 was upregulated in the AMI dataset while it was reported to be downregulated in the literature [22]. In UA, the regulation pattern of the overlapping miRNAs was found to be consistent between our study and the previous reported work [38].

### 3.3. Functional Enrichment Analysis of Target Genes of Candidate miRNA Biomarkers

**Figure 3:**
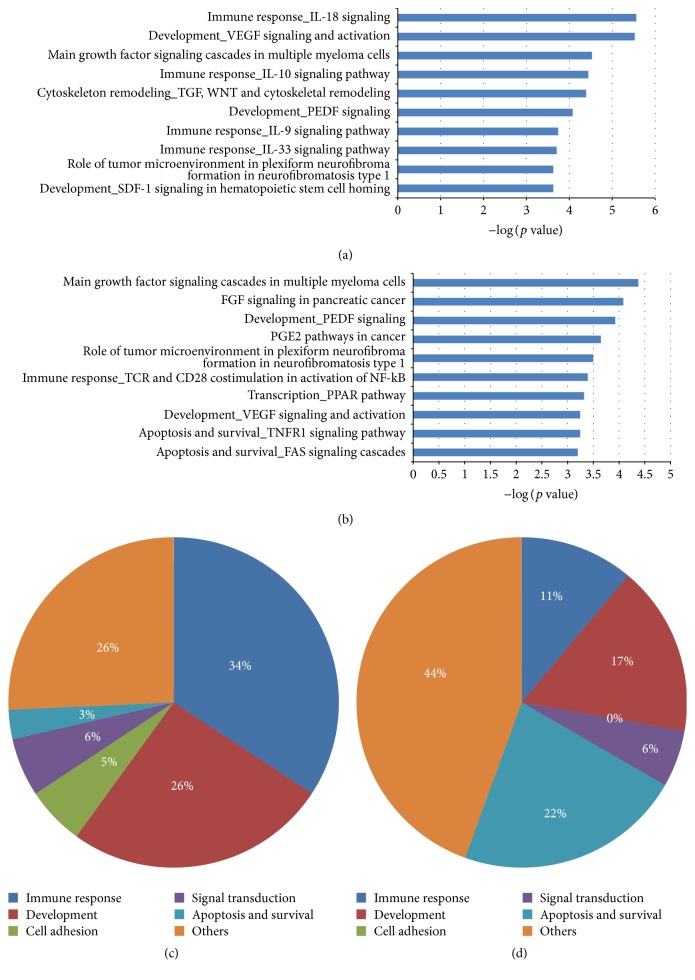
Significantly enriched pathways by targets of microRNA biomarkers for acute myocardial infarction (AMI) and unstable angina (UA). (a) Top 10 enriched pathways in AMI. (b) Top 10 enriched pathways in UA. (c) Pie plot of category enriched pathways in AMI. (d) Pie plot of category enriched pathways in UA.

We further explored the roles of uniquely regulated genes of the identified miRNAs in AMI and UA by functional enrichment analysis using the MetaCore software [39–44]. In pathway analysis, we found 35 significantly enriched pathways in AMI and 18 in UA (*p* value < 0.05 and FDR < 0.05; see Figures 3(a) and 3(b)). There were nine pathways significantly enriched by the targets of candidate miRNA biomarkers for both AMI and UA (see Tables S3 and S4).

In general, the significantly enriched pathways were grouped into immune response, development, cell adhesion, signal transduction, apoptosis, and survival, and others as shown in Figures 3(c) and 3(d). In AMI, pathways in immune response (34%) and development (26%) account for 60% of the pathways. In UA, immune response and developmental pathways also play a role, with 11% and 17% of the miRNA-regulated pathways belonging to these categories, respectively. Besides these two, apoptosis and survival pathways accounted for a combined 22% of all miRNA targets.

We then evaluated the relevance of these pathways in AMI and UA by searching PubMed for published papers describing the role of constituent network objects of pathways in AMI and UA. As shown in Table S3, 28 of the 35 AMI pathways were reported to be involved with AMI and 10 of them are in the group of immune responses, such as CD40 signaling [45, 46]. Many interleukin (IL) factors were also reported in immune response pathways related to AMI such as IL-9 [47], IL-10 [48], IL-17 [49], IL-18 [50], and IL-33 [51]. In the development group, there were five pathways related to AMI, including WNT [52], G-CSF [53], SDF-1 [54], NF-kB [55], PEDF [56], and VEGF [57].

In the 18 pathways found in UA, 12 had been reported previously to relate to UA. The most important pathways were involved with apoptosis and survival (FAS signaling cascades [58], TNFR1 signaling pathway [59], and NGF activation of NF-kB [60]). The other correlated pathways that had been previously reported were the PPAR pathway [61] and TCR and CD28 costimulation in activation of NF-kB [62] (see Table S4).

### 3.4. The Percentage of Pathways Potentially Regulated by Each Biomarker miRNA

Analyzing the biological processes for each subtype revealed mechanistic relationships. Some of the pathways may result in atherosclerosis progression and atherosclerotic lesion rupture. However, some may contribute to the development of coronary collateral vessels, and some may even have their roles in inhibiting the formation of the thrombus. In order to explore the role of miRNAs in the pathways, we calculated the percentage of pathways that were regulated by miRNA in all significantly enriched pathways (see Table 2).

In AMI, miR-126 (83%), let-7g (69%), and miR-155 (46%) were the top three miRNAs that regulated more than 30% of the significantly enriched pathways (as listed in Table 2). miR-126 (72%), let-7g (72%), and miR-34a (33%) were the top three miRNAs involved in UA. Both in AMI and in UA, let-7g and miR-126 regulated more than half of the pathways, which indicated that they were functionally important to both of the subtypes. This observation is helpful for understanding the molecular mechanisms of ACS common or specific to the AMI and UA subtypes.

## 4. Discussion

Understanding the mechanism and identifying the biomarkers specific to AMI and UA are important for ACS diagnosis and treatments. In this study, miRNA biomarkers are identified for AMI and UA using our improved POMA model. The model enriches fragile sites in the miRNA-mRNA network, focusing on miRNAs that regulate important genes for these disease subtypes. We defined measures NOD and TFP to quantify whether a miRNA could be a candidate biomarker. The former reflected the power of a miRNA to regulate genes independently whereas the latter indicated the potential of regulating TF genes. TF genes are chosen because TFs are important regulators and are kernels of many crucial biological processes. According to our previous studies [23, 24], biomarker miRNAs tended to have large NOD and TFP values. Based on the evidence, we identified 27 and 26 miRNAs as candidate biomarkers for AMI and UA, respectively, and nine of them were shared by the two ACS subtypes. Five AMI and five UA candidates had been previously reported as miRNA biomarkers.

In order to explore the roles of miRNAs in ACS, we performed a functional enrichment analysis for the targets of candidate miRNA biomarkers. In AMI, 35 pathways were significantly enriched and 28 (80%) have been reported to be related to AMI. Many of the pathways enriched in AMI were correlated with the immune response, and let-7g, miR-155, miR-101, miR-126, and miR-145 were closely relevant (see Table S3). A deregulated immune system is considered not only a trigger but also a factor amplifying an uncontrolled immune response in AMI [45]. CD40 signaling was reported to be upregulated in the pathogenesis of AMI patients [46]. The levels of IL-18 were upregulated in patients with AMI and the inhibition of its activity promoted cardiac function and reduced scar formation and infarct size [50, 63]. The predictive values of IL-6 and IL-10 were also shown for ST-elevation AMI [64]. IL-9 levels were significantly upregulated in patients with AMI compared with the stable angina pectoris and control groups [65]. IL-33/ST2 signaling is a mechanically activated, cardioprotective signaling system where IL-33 blocks angiotensin II- and phenylephrine-induced NF-*κ*B activation, and soluble ST2 inhibits the antihypertrophic effects of IL-33 [66]. The ratio of IL-33/sST2 also correlated with the 6-month prognosis of AMI patients [67]. Damaged myocardial tissue is repaired and replaced by scar tissue after MI, which triggers an inflammatory cascade. Clinical studies have indicated that an excessive inflammatory reaction may evoke adverse remodeling and directly affect prognosis in patients with AMI. It has been suggested that elevated concentrations of circulating neutrophils and monocytes and enhanced extracellular matrix breakdown [68] may contribute to infarct expansion or even cardiac rupture [69]. Accumulating evidence also showed that uncontrolled immune response in AMI may result from a pleiotropic proinflammatory imbalance [70]. Accordingly, exploring the DE miRNAs and target genes within these immune cells may be promising for cell base therapies.

In UA, 12 of 18 (66%) significantly enriched pathways were reported previously and 22% of the pathways were grouped in the apoptosis and survival category. Apoptosis is programmed cell death or physiological death. The abnormalities of apoptosis may contribute to plaque rupture and ACS. Endothelial cell apoptosis differs from macrophage/monocyte apoptosis [71]; endothelial cell apoptosis results in atherosclerosis progression and atherosclerotic lesion rupture [72]. The increased expression of Fas and FasL (both in AMI and in UA) was observed on the surface of peripheral blood lymphocytes [73, 74]. Cellular apoptosis may be one of the factors involved in atherosclerosis and may play a role in the rupture of atherosclerotic plaques. Thus, we not only should get a better understanding of the whole process of programmed cell death but also need to know the contribution of antiapoptotic therapy to plaque stabilization. More importantly, miRNAs like miR-346, miR-196b, miR-126, and let-7g were functional in these pathways according to our study (see Table S4), which indicates their potential roles in the process of cell apoptosis as well as the occurrence and progression of UA.

Moreover, there were nine pathways that were enriched by targets of miRNA biomarkers in both AMI and UA. Two developmental signaling pathways, vascular endothelial growth factor (VEGF) and pigment epithelium-derived factor (PEDF), were reported to be important factors in both subtypes. VEGF, a peripheral blood cytokine, is mainly derived from platelets and granulocytes and in particular neutrophils, which play a crucial role in vascular formation in physiological and pathological conditions [75–77]. It has been reported that, in ischemic conditions, VEGF promotes the development of coronary collateral vessels, providing adequate blood supply and preventing death of cardiomyocytes [78]. Many studies have found that serum VEGF concentrations were elevated in ACS, which can be a surrogate marker of myocardial infarction [79, 80]. Serum VEGF-A was shown to be elevated after AMI, which suggested a role for the formation of coronary collateral vessels [57, 81]. VEGF-A is also a target gene of miR-126, which mapped in the pathway.

The other common developmental pathway in AMI and UA was PEDF signaling. PEDF, a 50-kDa glycoprotein, has anti-inflammatory, antioxidant, antiangiogenic, antithrombotic, antitumorigenic, and neuroprotective properties [82] and is widely expressed throughout the human body. In ACS patients, plasma PEDF concentrations were significantly lower than the control group and associated with adverse cardiac outcomes after ACS [83]. PEDF can block platelet activation and aggregation [84] through its anti-inflammatory and antioxidative properties, leading to the inhibition of the vascular inflammation and the formation of a thrombus [85].

We also calculated the percentage of pathways that were potentially regulated by the miRNAs in all significantly enriched pathways (see Tables 2, S3, and S4). A novel miRNA (let-7g), which had never been reported as an important factor in ACS before, deserves further investigation, as it participated in regulating 69% (24/35) of the pathways in AMI and 72% (13/18) in UA. About half of these enriched pathways were closely associated with the immune response, especially in AMI (see Tables S3 and S4). Notably, it was previously reported that a miRNA together with its targets was differentially regulated in E2F1-deficient mice, and the E2F1 transcription factor played important roles in the immune response to systemic* Escherichia coli* lipopolysaccharide (LPS) [86]. The conclusions demonstrated the significance of let-7g in the immune response, which may represent a latent therapeutic target for the treatment of immunological diseases as well as ACS. More clinical validations of this hypothesis will be needed in the future.

We noticed that the miRNA datasets selected for our comparison in this study were inconsistent. The AMI dataset was obtained on whole blood (GSE31568) whereas the UA was on plasma (GSE49823). As we known, the concentration of miRNAs in whole blood is higher than that in plasma; thus the prediction based on the two data sources has some limitations. It would be better if we could obtain AMI and UA samples chosen from the same source type (both were from plasma or whole blood) and compare with the same control group. Unfortunately, the miRNA expression data that could be used for analyses were quite limited. On the other hand, we considered that plasma is an important component of whole blood, where miRNAs could present in a remarkably stable form [87]. Hence further expression data analyses and clinical validations need to be done when more and better datasets are available for in-depth studies.

## 5. Conclusions

In this study, we applied our improved POMA model to identify miRNA biomarkers for subtyping ACS, finding 18 and 17 miRNAs to be specific biomarkers for AMI and UA, respectively. Nine miRNAs were found in both subtypes, which implied that they could be universal molecular markers for ACS. These findings were further verified by enrichment analysis and compared with previous publications. For future translational application, further experimental and clinical verifications are necessary.

## Supplementary Material

TABLE S1: Literature reported miRNA biomarkers for acute myocardial infarction (AMI). TABLE S2: Literature reported miRNA biomarkers for unstable angina (UA). TABLE S3: Significantly enriched pathways by targets of candidate biomarker miRNAs for acute myocardial infarction (AMI). TABLE S4: Significantly enriched pathways by targets of candidate biomarker miRNAs for unstable angina (UA).

## Figures and Tables

**Table 1:** Summary of the miRNA datasets used in this study.

Subtype	GEO accession	Platform	Number of probes	Number of samples (control/disease)
AMI	GSE 31568	Febit Homo Sapiens miRBase 13.0	866	91 (70/21)
UA	GSE 49823	TaqMan® Human MiRNA Array v3.0 A/B	768	26 (13/13)

**Table 2:** The identified miRNA biomarker candidates for acute myocardial infarction (AMI) and unstable angina (UA).

AMI					UA				
miRNA ID	*N*	NOD	TF (TFP)	Pathways (percentage)	miRNA ID	*N*	NOD	TF (TFP)	Pathways (percentage)
**miR-155**	185	64	39 (0.21)	16 (0.46)	miR-197	151	32	24 (0.16)	2 (0.11)
miR-30e	356	32	56 (0.16)	5 (0.14)	miR-125b	109	30	20 (0.18)	4 (0.22)
miR-98	329	24	62 (0.19)	2 (0.06)	miR-590-3p	255	18	44 (0.17)	2 (0.11)
miR-23b	211	18	34 (0.16)	5 (0.14)	miR-22^*∗*^	158	16	32 (0.20)	0
miR-204	198	15	40 (0.20)	10 (0.29)	miR-204	198	15	40 (0.20)	2 (0.11)
**miR-34a**	80	14	15 (0.19)	5 (0.14)	miR-34a	80	14	15 (0.19)	6 (0.33)
let-7g	199	13	34 (0.17)	24 (0.69)	miR-486-3p	152	13	23 (0.15)	2 (0.11)
miR-576-3p	133	13	23 (0.17)	0	let-7g	199	13	34 (0.17)	13 (0.72)
miR-346	31	13	5 (0.16)	5 (0.14)	miR-346	31	13	5 (0.16)	4 (0.22)
miR-454	298	13	43 (0.14)	0	miR-340	256	11	37 (0.14)	1 (0.06)
miR-532-3p	112	12	18 (0.16)	5 (0.14)	miR-340 ^*∗*^	256	11	37 (0.14)	1 (0.06)
miR-145	55	11	11 (0.20)	8 (0.23)	miR-196b	165	11	27 (0.16)	5 (0.28)
miR-340 ^*∗*^	256	11	37 (0.14)	3 (0.09)	miR-145	55	11	11 (0.20)	3 (0.17)
**miR-126**	34	10	5 (0.15)	29 (0.83)	miR-324-3p	84	10	12 (0.14)	1 (0.06)
miR-621	65	10	13 (0.20)	9 (0.26)	**miR-126**	34	10	5 (0.15)	13 (0.72)
miR-142-3p	87	8	18 (0.21)	3 (0.09)	**miR-106b**	376	9	61 (0.16)	1 (0.06)
miR-31	34	7	8 (0.24)	5 (0.14)	miR-885-5p	89	9	14 (0.16)	0
miR-600	127	7	23 (0.18)	1 (0.03)	**miR-132**	46	8	7 (0.15)	0
miR-491-3p	119	6	21 (0.18)	0	miR-17	80	8	13 (0.16)	5 (0.28)
miR-603	149	6	32 (0.21)	2 (0.06)	miR-597	76	8	12 (0.16)	2 (0.11)
miR-93	394	6	68 (0.17)	0	miR-142-3p	87	8	18 (0.21)	1 (0.06)
miR-934	72	5	12 (0.17)	0	**miR-25**	260	7	40 (0.15)	0
**miR-27a**	50	5	15 (0.30)	3 (0.09)	**miR-590-5p**	112	7	23 (0.21)	0
miR-548b-3p	103	5	18 (0.17)	0	miR-324-5p	78	7	18 (0.23)	2 (0.11)
**miR-101**	69	4	18 (0.26)	4 (0.11)	miR-93	394	6	68 (0.17)	0
miR-128	22	4	4 (0.18)	0	miR-618	112	6	17 (0.15)	0
miR-95	69	4	10 (0.14)	0					

*Notes. *The miRNAs were ranked based on their NOD values. miRNA IDs in bold have been reported in published studies and those with underlines were shared by both AMI and UA.

## Abbreviations

DE: Differentially expressed
TF: Transcription factor
POMA: Pipeline of Outlier MicroRNA Analysis
NOD: Novel out degree
TFP: Transcription factor gene percentage.
