# Supplementary material for: Novel Biomarker MicroRNAs for Subtyping of Acute Coronary Syndrome: A Bioinformatics Approach
Source: Biomed Res Int. 2016 Dec 1;2016:4618323. doi: 10.1155/2016/4618323 (PMC5156791; doi:10.1155/2016/4618323)
Supplement: Supplementary file 1 — TABLE S1: Literature reported miRNA biomarkers for acute myocardial infarction (AMI). TABLE S2: Literature reported miRNA biomarkers for unstable angina (UA). TABLE S3: Significantly enriched pathways by targets of candidate biomarker miRNAs for acute myocardial infarction (AMI). TABLE S4: Significantly enriched pathways by targets of candidate biomarker miRNAs for unstable angina (UA). [file 4618323.f1.doc]

**Supplementary Materials**

**TABLE S1:** Literature reported miRNA biomarkers for acute myocardial infarction (AMI).

| **miRNA ID** | **Type** | **Expression Pattern** | **Source** | **Control** | **Cases** | **District** | **Year** | **PMID** | **Experiment method** |
| --- | --- | --- | --- | --- | --- | --- | --- | --- | --- |
| miR-1 | Diagnosis | Up | Plasma | 327 AMI | 117 UA | Germany | 2011 | 21806992 | qRT-PCR |
|  | Diagnosis | Up | Plasma | 25 healthy | 17 AMI | China | 2012 | 22719221 | RT-PCR |
|  | Diagnosis | Up | Plasma | 159 healthy | 159 AMI | China | 2010 | 19896465 | RT-PCR |
|  | Diagnosis | Up | Plasma | 28 non-AMI | 56 AMI | China | 2014 | 25225581 | qRT-PCR |
|  | Diagnosis | Up | Plasma | 32 healthy | 67 AMI | China | 2013 | 23420161 | qRT-PCR |
|  | Diagnosis | Up | Serum | 100 heathy | 117 AMI | China | 2013 | 23641832 | RT-qPCR |
|  | Diagnosis | Up | Plasma | 17 healthy | 33 STEMI | Italy | 2010 | 20534597 | RT-qPCR |
| miR-1291 | Diagnosis | Up | Plasma | 110 non-AMI | 76 AMI | China | 2014 | 24885383 | qRT-PCR |
|  | Diagnosis | Up | Blood | 20 healthy | 20 AMI | Germany | 2011 | 20886220 | qRT-PCR |
| miR-133 | Diagnosis | Up | Plasma | 28 healthy | 51 AMI | China | 2011 | 21881276 | qRT-PCR |
|  | Diagnosis | Up | Plasma | 110 non-AMI | 76 AMI | China | 2014 | 24885383 | qRT-PCR |
| miR-133a | Diagnosis | Up | Plasma | 327 AMI | 117 UA | Germany | 2011 | 21806992 | qRT-PCR |
|  | Prognosis |  |  |  |  |  |  |  |  |
|  | Diagnosis | Up | Plasma | 127 healthy | 13 AMI | China | 2013 | 24053180 | qRT-PCR |
|  | Diagnosis | Up | Plasma | 32 healthy | 67 AMI | China | 2013 | 23420161 | qRT-PCR |
|  | Diagnosis | Up | Plasma | 17 healthy | 33 STEMI | Italy | 2010 | 20534597 | RT-qPCR |
| miR-133b | Diagnosis | NA | Plasma | 327 AMI | 117 UA | Germany | 2011 | 21806992 | qRT-PCR |
|  | Diagnosis | Up | Plasma | 17 healthy | 33 STEMI | Italy | 2010 | 20534597 | RT-qPCR |
| miR-134 | Diagnosis | Up | Plasma | 30 healthy | 359 AMI | China | 2014 | 24833470 | qRT-PCR |
|  | Diagnosis | Up | Serum | 100 heathy | 117 AMI | China | 2013 | 23641832 | RT-qPCR |
| miR-155 | Prognosis | Up | Serum | 28 Survival (AMI) | 26 Cardiac death (AMI) | Japan | 2012 | 22995291 | qRT-PCR |
| miR-186 | Diagnosis | Up | Serum | 100 heathy | 117 AMI | China | 2013 | 23641832 | RT-qPCR |
| miR-192 | Diagnosis | Up | Serum | 65 AMI | 65AMI（HF） | Japan | 2013 | 23743335 | NA |
| miR-194 | Diagnosis | Up | Serum | 65 AMI | 65AMI（HF） | Japan | 2013 | 23743335 | NA |
| miR-195 | Diagnosis | Up | Plasma | 30 healthy | 18 AMI | China | 2012 | 23236408 | qRT-PCR |
| miR-208 | Diagnosis | Up | serum | 100 heathy | 117 AMI | China | 2013 | 23641832 | RT-qPCR |
| miR-208a | Diagnosis | NA | Plasma | 327 AMI | 117 UA | Germany | 2011 | 21806992 | qRT-PCR |
|  | Diagnosis | Up | Plasma | 30 healthy | 33 AMI | China | 2010 | 20159880 | RT-PCR |
| miR-208b | DiagnosisPrognosis | Up | Plasma | 327 AMI | 117 UA | Germany | 2011 | 21806992 | qRT-PCR |
|  | Diagnosis | Up | Plasma | 32 healthy | 67 AMI | China | 2013 | 23420161 | qRT-PCR |
| miR-223 | Diagnosis | Up | Serum | 100 heathy | 117 AMI | China | 2013 | 23641832 | RT-qPCR |
| miR-328 | Diagnosis | Up | Plasma | 30 healthy | 359 AMI | China | 2014 | 24833470 | qRT-PCR |
|  | Diagnosis | Up | Plasma | 28 healthy | 51 AMI | China | 2011 | 21881276 | qRT-PCR |
| miR-30a | Diagnosis | Up | Plasma | 30 healthy | 18 AMI | China | 2012 | 23236408 | qRT-PCR |
| miR-34a | Diagnosis | Up | Serum | 65 AMI | 65AMI（HF） | Japan | 2013 | 23743335 | NA |
| miR-361-5p | Diagnosis | Up | Blood | 28 healthy | 17 AMI | China | 2014 | 25184815 | qRT-PCR |
| miR-380* | Prognosis | Up | Serum | 28 Survival (AMI) | 26 Cardiac death (AMI) | Japan | 2012 | 22995291 | qRT-PCR |
| miR-423-5p | Diagnosis | Up | Plasma | 5 healthy | 17 AMI | Poland | 2013 | 24253456 | TaqMan® MicroRNA Assays |
| miR-497 | Diagnosis | Up | Blood | 31 healthy | 27 AMI | China | 2014 | 25110754 | qRT-PCR |
| miR-499 | Diagnosis | NA | Plasma | 327 AMI | 117 UA | Germany | 2011 | 21806992 | qRT-PCR |
|  | Diagnosis | Up | Plasma | 32 healthy | 67 AMI | China | 2013 | 23420161 | qRT-PCR |
|  | Diagnosis | Up | Plasma | 10 heathy | 29(ACS+CHF) | Japan | 2010 | 20395621 | RT-PCR |
|  | Diagnosis | Up | Serum | 100 heathy | 117 AMI | China | 2013 | 23641832 | RT-qPCR |
|  | Diagnosis | Up | Serum | 87 healthy | 510 MI | Luxembourg | 2012 | 22252325 | RT-qPCR |
| miR-499-5p | Diagnosis | Up | Plasma | 17 healthy | 33 AMI(STEMI) | Italy | 2010 | 20534597 | RT-qPCR |
|  | Diagnosis | Up | Plasma | 99 healthy | 92 AMI(NSTEMI) | Italy | 2013 | 22330002 | qRT-PCR |
| miR-122 | Diagnosis | Down | Plasma | 17 healthy | 33 AMI(STEMI) | Italy | 2010 | 20534597 | RT-PCR |
| miR-126 | Diagnosis | Down | Plasma | 25 healthy | 17 AMI | China | 2012 | 22719221 | qRT-PCR |
| miR-375 | Diagnosis | Down | Plasma | 17 healthy | 33AMI(STEMI) | Italy | 2010 | 20534597 | RT-PCR |
| miR-519e-5p | Diagnosis | Down | Blood | 28 healthy | 17 AMI | China | 2014 | 25184815 | qRT-PCR |
| let-7b | Diagnosis | Down | Plasma | 30 healthy | 18 AMI | China | 2012 | 23236408 | qRT-PCR |
| miR-16/27a/  101/150 | Prognosis | Down: miR-150  miR-101 | Plasma | 79 WMIS<= 1.2 | 71 WMIS> 1.2 | UK | 2013 | 23967079 | qPCR |
|  |  | Up:  miR-16  miR-27a |  |  |  |  |  |  |  |

*Note:* Here, “NA” is the abbreviation of “not available”.

**TABLE S2: Literature reported miRNA biomarkers for unstable angina (UA).**

| **miRNA ID** | **Type** | **Expression Pattern** | **Source** | **Control** | **Cases** | **District** | **Year** | **PMID** | **Experiment method** |
| --- | --- | --- | --- | --- | --- | --- | --- | --- | --- |
| miR-106b | Diagnosis | Up | Plasma | 5 healthy | 5 UA | China | 2013 | 24339880 | qRT-PCR |
| miR-1 | Diagnosis | Up | Plasma | 20 healthy | 19 UA | Italy | 2013 | 24260372 | qRT-PCR |
|  | Diagnosis | Down | Plasma | 327 AMI | 117 UA | Germany | 2011 | 21806992 | qRT-PCR |
| miR-126 | Diagnosis | Up | Plasma | 20 healthy | 19 UA | Italy | 2013 | 24260372 | qRT-PCR |
| miR-126* | Diagnosis | Up | Plasma | 5 healthy | 5 UA | China | 2013 | 24339880 | qRT-PCR |
| miR-133a | Diagnosis | Up | Plasma | 20 healthy | 19 UA | Italy | 2013 | 24260372 | qRT-PCR |
|  | DiagnosisPrognosis | Down | Plasma | 327 AMI | 117 UA | Germany | 2011 | 21806992 | qRT-PCR |
| miR-133b | Diagnosis | NA | Plasma | 327 AMI | 117 UA | Germany | 2011 | 21806992 | qRT-PCR |
| miR-208a | Diagnosis | NA | Plasma | 327 AMI | 117 UA | Germany | 2011 | 21806992 | qRT-PCR |
| miR-208b | DiagnosisPrognosis | Down | Plasma | 327 AMI | 117 UA | Germany | 2011 | 21806992 | qRT-PCR |
| miR-21 | Diagnosis | Up | Plasma | 5 healthy | 5 UA | China | 2013 | 24339880 | qRT-PCR |
| miR-25 | Diagnosis | Up | Plasma | 5 healthy | 5 UA | China | 2013 | 24339880 | qRT-PCR |
| miR-451 | Diagnosis | Up | Plasma | 5 healthy | 5 UA | China | 2013 | 24339880 | qRT-PCR |
| miR-499 | Diagnosis | Up | Plasma | 327 AMI | 117 UA | Germany | 2011 | 21806992 | qRT-PCR |
| miR-590-5p | Diagnosis | Up | Plasma | 5 healthy | 5 UA | China | 2013 | 24339880 | qRT-PCR |
| miR-92a | Diagnosis | Up | Plasma | 5 healthy | 5 UA | China | 2013 | 24339880 | qRT-PCR |
| miR-132/150/186 | Diagnosis | NA | Serum | 120 NCCP | 104 UA | Germany | 2014 | 24727883 | qRT-PCR |
|  |  |  |  | 20 healthy |  |  |  |  |  |

*Note:* Here, “NA” is the abbreviation of “not available”.

**TABLE S3:** Significantly enriched pathways by targets of candidate biomarker miRNAs for acute myocardial infarction (AMI).

| **Enriched pathway** | **p-value** | **FDR** | **Ratio** | **miRNA ID** | **PMID** |
| --- | --- | --- | --- | --- | --- |
| Immune response_IL-18 signaling | 2.75E-06 | 8.81E-04 | 8/60 | **let-7g**,miR-204,miR-346,miR-101,miR-126,miR-31,miR-155 | 24389343 |
| Development_VEGF signaling and activation | 2.99E-06 | 8.81E-04 | 7/43 | miR-155,miR-27a,**let-7g**,miR-126,miR-31 | 23313225  15345590 |
| Main growth factor signaling cascades in multiple myeloma cells | 2.97E-05 | 4.79E-03 | 6/41 | miR-126,**let-7g** | NA |
| Immune response_IL-10 signaling pathway | 3.60E-05 | 4.79E-03 | 7/62 | miR-532-3p,miR-204,miR-145,  miR-155,**let-7g**,miR-126,miR-101 | 22931953 |
| Cytoskeleton remodeling_TGF, WNT and cytoskeletal remodeling | 4.06E-05 | 4.79E-03 | 9/111 | miR-532-3p,miR-98,miR-31,**let-7g**,miR-126,miR-34a,miR-603 | 10728347 |
| Development_PEDF signaling | 8.36E-05 | 8.20E-03 | 6/49 | miR-34a,miR-126,miR-346 | 24192856  21281791  22315956 |
| Immune response_IL-9 signaling pathway | 1.82E-04 | 1.40E-02 | 5/36 | miR-30e,miR-145,**let-7g**,miR-621,miR-126 | 24453425 |
| Immune response_IL-33 signaling pathway | 1.97E-04 | 1.40E-02 | 6/57 | miR-155,miR-145,miR-31,miR-126,miR-621 | 17492053  24112154  24837094  25458175 |
| Role of tumor microenvironment in plexiform neurofibroma formation in neurofibromatosis type 1 | 2.37E-04 | 1.40E-02 | 5/38 | **let-7g**,miR-126 | 9313104  11144803 |
| Development_SDF-1 signaling in hematopoietic stem cell homing | 2.37E-04 | 1.40E-02 | 5/38 | **let-7g**,miR-126,miR-204,miR-23b | 15992820 |
| Development_c-Kit ligand signaling pathway during hemopoiesis | 2.86E-04 | 1.53E-02 | 6/61 | miR-126,miR-204,miR-145,**let-7g**,miR-23b | NA |
| Development_Role of IL-8 in angiogenesis | 4.06E-04 | 1.84E-02 | 6/65 | miR-532-3p,miR-126,miR-340* | 26550160 |
| Muscle contraction_Regulation of eNOS activity in endothelial cells | 4.06E-04 | 1.84E-02 | 6/65 | miR-621,miR-126,miR-27a,  miR-600,miR-155 | 9740620 |
| Immune response_TNF-R2 signaling pathways | 5.30E-04 | 2.16E-02 | 5/45 | miR-34a,miR-621,miR-126 | 19576194  10591022 |
| FGF signaling in pancreatic cancer | 5.88E-04 | 2.16E-02 | 5/46 | miR-126,**let-7g**,miR-204,miR-155 | 21416207  11834506 |
| Immune response_MIF-induced cell adhesion, migration and angiogenesis | 5.88E-04 | 2.16E-02 | 5/46 | miR-126,**let-7g**,miR-204 | 12704210 |
| Development_G-CSF signaling | 7.89E-04 | 2.45E-02 | 5/49 | miR-155,miR-126,miR-145,**let-7g** | 15992820 |
| Development_ThromboxaneA2 signaling pathway | 7.89E-04 | 2.45E-02 | 5/49 | miR-155,**let-7g**,miR-142-3p,  miR-126 | 17196455  23909754 |
| Tissue Factor signaling in cancer via PAR1 and PAR2 | 7.89E-04 | 2.45E-02 | 5/49 | miR-126,miR-23b,miR-340*,**let-7g** | 22518344 |
| Apoptosis and survival_NGF activation of NF-kB | 8.57E-04 | 2.52E-02 | 4/29 | **let-7g**,miR-155,miR-126 | 24508054  22628392  22001051 |
| Signal transduction_NF-kB activation pathways | 9.49E-04 | 2.66E-02 | 5/51 | miR-34a,miR-145,miR-155,miR-621 | 18611340  14676146 |
| Development_WNT signaling pathway. Part 2 | 1.13E-03 | 2.90E-02 | 5/53 | miR-31,miR-34a,miR-603,miR-155,miR-126 | 22173911  22085926 |
| Signal transduction_Additional pathways of NF-kB activation (in the cytoplasm) | 1.13E-03 | 2.90E-02 | 5/53 | miR-621,miR-155,miR-126,  miR-142-3p,**let-7g** | 25409294 |
| Cell adhesion_Alpha-4 integrins in cell migration and adhesion | 1.58E-03 | 3.72E-02 | 4/34 | miR-532-3p,miR-126 | NA |
| Development_CNTF receptor signaling | 1.58E-03 | 3.72E-02 | 4/34 | miR-30e,miR-126,miR-145,**let-7g** | 22507542 |
| Cell adhesion_Tight junctions | 1.96E-03 | 4.02E-02 | 4/36 | miR-155,miR-98,miR-30e,miR-23b | 25768344 |
| HBV signaling via protein kinases leading to HCC | 1.96E-03 | 4.02E-02 | 4/36 | miR-155,miR-204,**let-7g**,miR-23b | NA |
| Immune response_Regulation of T cell function by CTLA-4 | 1.96E-03 | 4.02E-02 | 4/36 | miR-155,**let-7g**,miR-346,miR-126 | 17652883 |
| Immune response_IL-17 signaling pathways | 1.98E-03 | 4.02E-02 | 5/60 | miR-101,miR-126,miR-204,  miR-621 | 22956509  18294918 |
| Immune response_Oncostatin M signaling via MAPK in human cells | 2.17E-03 | 4.26E-02 | 4/37 | miR-30e,miR-145,miR-204,**let-7g** | 22056139 |
| Regulation of lipid metabolism_Regulation of lipid metabolism via LXR, NF-Y and SREBP | 2.40E-03 | 4.56E-02 | 4/38 | miR-532-3p,miR-155 | NA |
| Immune response_IL-15 signaling | 2.64E-03 | 4.85E-02 | 5/64 | miR-30e,miR-126,**let-7g**,miR-621 | 26275688 |
| Immune response_CD40 signaling | 2.82E-03 | 4.89E-02 | 5/65 | miR-126,miR-621,miR-346,**let-7g**,miR-101 | 14769218 |
| Immune response_TCR and CD28 co-stimulation in activation of NF-kB | 2.90E-03 | 4.89E-02 | 4/40 | miR-346,miR-126,**let-7g**,miR-340* | NA |
| Reproduction_Progesterone-mediated oocyte maturation | 2.90E-03 | 4.89E-02 | 4/40 | miR-204,miR-142-3p,**let-7g**,  miR-27a | NA |

*Note:* Underlined pathways are regulated by biomarker miRNAs that were enriched in both AMI and UA. Here, “NA” is the abbreviation of “not available”.

**TABLE S4: Significantly enriched pathways by targets of candidate biomarker miRNAs for unstable angina (UA).**

| **Enriched pathway** | **p-value** | **FDR** | **Ratio** | **miRNA ID** | **PMID** |
| --- | --- | --- | --- | --- | --- |
| Main growth factor signaling cascades in multiple myeloma cells | 4.23E-05 | 2.34E-02 | 6/41 | miR-126,**let-7g** | 25512274 |
| FGF signaling in pancreatic cancer | 8.26E-05 | 2.34E-02 | 6/46 | miR-590-3p,miR-17,miR-126,**let-7g**,miR-204 | NA |
| Development_PEDF signaling | 1.19E-04 | 2.34E-02 | 6/49 | miR-34a,miR-126,miR-346 | 25356816 |
| PGE2 pathways in cancer | 2.27E-04 | 3.09E-02 | 6/55 | miR-590-3p,miR-597,miR-126,miR-142-3p,**let-7g**,miR-34a,miR-486-3p | 3529901 |
| Role of tumor microenvironment in plexiform neurofibroma formation in neurofibromatosis type 1 | 3.18E-04 | 3.09E-02 | 5/38 | **let-7g**,miR-126 | NA |
| Immune response_TCR and CD28 co-stimulation in activation of NF-kB | 4.06E-04 | 3.09E-02 | 5/40 | miR-346,miR-486-3p,miR-196b,miR-126,**let-7g**,miR-340,  miR-340* | 16139128 |
| Transcription_PPAR Pathway | 4.80E-04 | 3.09E-02 | 6/63 | **let-7g**,miR-126,miR-324-5p | 25604313 |
| Apoptosis and survival_TNFR1 signaling pathway | 5.72E-04 | 3.09E-02 | 5/43 | miR-34a,miR-196b,miR-346,miR-125b | 21039303 |
| Development_VEGF signaling and activation | 5.72E-04 | 3.09E-02 | 5/43 | miR-126,**let-7g**,miR-196b | 12566361 |
| Apoptosis and survival_FAS signaling cascades | 6.37E-04 | 3.09E-02 | 5/44 | miR-145,miR-34a,miR-125b,miR-346 | 17853317 |
| Ligand-independent activation of Androgen receptor in Prostate Cancer | 6.69E-04 | 3.09E-02 | 6/67 | miR-17,**let-7g**,miR-125b,  miR-126 | NA |
| Regulation of GSK3 beta in bipolar disorder | 7.07E-04 | 3.09E-02 | 5/45 | miR-324-3p,miR-197,**let-7g**,  miR-34a,miR-17 | NA |
| Apoptosis and survival_NGF signaling pathway | 7.09E-04 | 3.09E-02 | 4/26 | miR-145,**let-7g**,miR-126,  miR-17 | 25512274 |
| Development_TGF-beta-dependent induction of EMT via RhoA, PI3K and ILK. | 7.83E-04 | 3.09E-02 | 5/46 | **let-7g**,miR-106b,miR-126,  miR-17 | NA |
| Immune response_MIF-induced cell adhesion, migration and angiogenesis | 7.83E-04 | 3.09E-02 | 5/46 | miR-204,**let-7g**,miR-126 | 25821795  20934703 |
| Apoptosis and survival_NGF activation of NF-kB | 1.09E-03 | 4.01E-02 | 4/29 | **let-7g**,miR-196b,miR-126,  miR-597 | 9788823 |
| Regulation of lipid metabolism_RXR-dependent regulation of lipid metabolism via PPAR, RAR and VDR | 1.24E-03 | 4.13E-02 | 4/30 | miR-324-5p,miR-125b | NA |
| Signal transduction_NF-kB activation pathways | 1.26E-03 | 4.13E-02 | 5/51 | miR-34a,miR-196b,miR-145,miR-197 | 9788823 |

*Note:* Underlined pathways are regulated by biomarker miRNAs that were enriched in both AMI and UA. Here, “NA” is the abbreviation of “not available”.
